# Are We Underestimating Zygomaticus Variability in Midface Surgery?

**DOI:** 10.3390/jcm14207311

**Published:** 2025-10-16

**Authors:** Ingrid C. Landfald, Łukasz Olewnik

**Affiliations:** 1 Department of Clinical Anatomy, Mazovian Academy in Plock, 09-402 Płock, Poland; ingridceciliee@gmail.com; 2 VARIANTIS Research Laboratory, 09-409 Płock, Poland

**Keywords:** zygomaticus major, zygomaticus minor, anatomical variation, Landfald classification, dynamic ultrasound, EMG mapping, facelift, smile reconstruction

## Abstract

The zygomaticus major and minor (ZMa/ZMi) are key determinants of smile dynamics and midface contour, yet they exhibit substantial morphological variability—including bifid or multibellied bellies, accessory slips, and atypical insertions. Such variants can alter force vectors, fat-compartment boundaries, and SMAS planes, increasing the risk of asymmetry, contour irregularities, or “joker smile” following facelifts, fillers, thread lifts, and smile reconstruction. To our knowledge, this is the first review to integrate the Landfald classification of ZMa/ZMi variants with a standardized dynamic imaging-based workflow for aesthetic and reconstructive midface procedures. We conducted a narrative literature synthesis of anatomical and imaging studies. Bifid or multibellied variants have been reported in up to 35% of cadaveric specimens. We synthesize anatomical, biomechanical, and imaging evidence (MRI, dynamic US, 3D analysis) to propose a practical protocol: (1) focused history and dynamic examination, (2) US/EMG mapping of contraction vectors, (3) optional high-resolution MRI for complex cases, and (4) individualized adjustment of surgical vectors, injection planes, and dosing. Procedure-specific adaptations are outlined for deep-plane releases, thread-lift trajectories, filler depth selection, and muscle-transfer orientation. We emphasize that standardizing preoperative dynamic mapping and adopting a “patient-specific mimetic profile” can enhance safety, predictability, and preservation of authentic expression, ultimately improving patient satisfaction across diverse midface interventions.

## 1. Introduction

### 1.1. Significance of Zygomatic Muscles in Midface Aesthetics and Expression

The zygomaticus major (ZMa) and minor (ZMi) are central to midface contour, malar projection, and a youthful, vibrant appearance. Their coordinated activity produces midface fullness and dynamic expressions, notably the smile, which strongly influences perceived attractiveness [1,2,3,4,5,6,7]. The ZMa elevates and laterally draws the oral commissure, while the ZMi subtly lifts the upper lip, adding complexity to facial movement.

**Figure 1:**
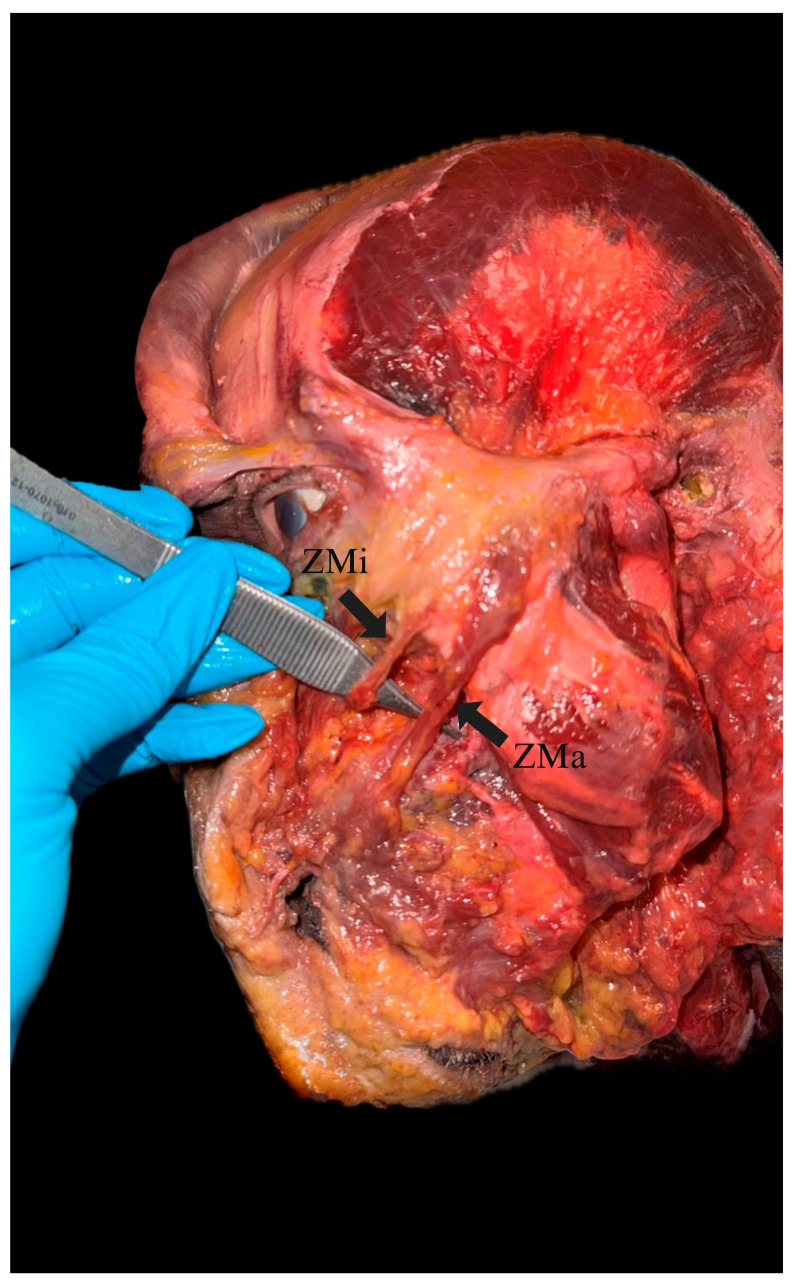
Cadaveric dissection of the left hemiface (ZMa/ZMi).

From an aesthetic perspective, an attractive smile requires both static harmony and dynamic balance. Symmetrical, natural activation especially of the ZMa is critical for an authentic “Duchenne smile,” associated with genuine happiness and positive social perception [8,9,10]. Variations such as bifid or accessory bands may disrupt this balance, leading to asymmetry or unnatural facial dynamics, underscoring the importance of precise preoperative assessment [11,12]—Figure 1.

### 1.2. Role as Functional Tension Vectors

Acting as key vectors of tension, the ZMa and ZMi influence skin positioning, symmetry, and the biomechanics of midfacial movement [1,2,12]. Understanding their directional forces is critical in facelifts, midface lifts, and volumetric procedures such as dermal fillers or autologous fat transfer [3,5]. Misinterpretation of their anatomy can result in inappropriate vectoring, leading to unnatural animation, compromised aesthetics, and patient dissatisfaction [2,5].

### 1.3. Aim and Scope of This Review

**Figure 2:**
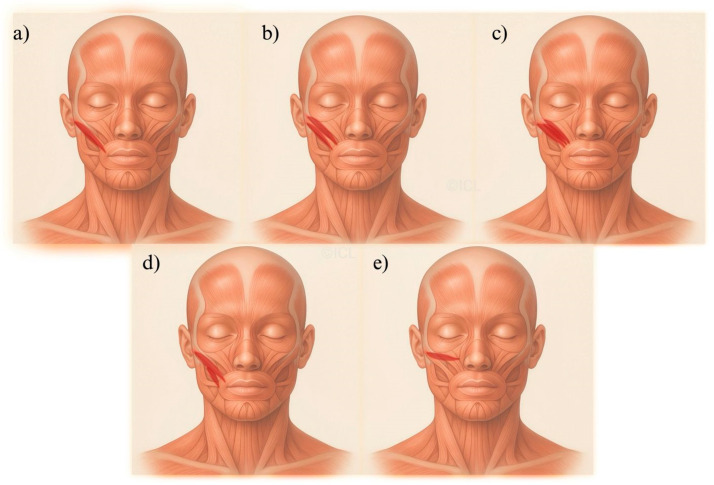
Schematic key to the Landfald–Olewnik five-type classification of the zygomaticus muscles. Arrows indicate dominant contraction vectors; solid dots mark muscle origins; open triangles mark insertions; and dashed lines outline clinically relevant SMAS/fat-compartment boundaries where applicable. Panels (**a**–**e**) correspond to Types I–V used throughout the text. (**a**) Type I—single belly (typical anatomy). ZMa: single fusiform belly inserting at/near the modiolus; stable, predictable lift vector. ZMi: slender, standard course with a high insertion to the upper lip serving as a reference pattern. (**b**) Type II—double belly/accessory fascicles. ZMa: bifid (superior/inferior) or accessory slips blending variably with the modiolus/LLS; dual vectors. ZMi: often more robust; may show blending with adjacent elevators, subtly altering vector balance and increasing asymmetry risk. (**c**) Type III—multibellied ZMa/high-riding ZMi. ZMa: >2 distinct bellies, increased vector complexity. ZMi: tends to insert higher and/or more laterally (“high-riding”) with variable insertion; superior vector dominance accentuates upper-lip pull. (**d**) Type IV—accessory bands/interdigitation. ZMa and ZMi: additional slips and interdigitations near the modiolus; includes ZMi duplicatus or crossing bands that modulate force transmission. ZMi may interdigitate with ZMa, making smile shape highly sensitive to minor tension changes; risk of dimpling/step-offs after aggressive release or thread paths. (**e**) Type V—atypical insertions. ZMa may be hypoplastic or variably inserting. ZMi: atypical attachments (e.g., upper lip, orbicularis oris, direct dermal insertions) that modify commissure elevation and smile dynamics; standard lift vectors may underperform, requiring vector re-direction or staged approaches.

This review highlights the clinical significance of ZMa and ZMi variability, integrating anatomical, biomechanical, and imaging data from plastic surgery, aesthetic medicine, neurology, and functional anatomy. To our knowledge, this is the first synthesis to link the Landfald–Olewnik classification of morphological variants with dynamic imaging-based preoperative planning. We discuss how these variants influence aesthetic outcomes, facial expression, and reconstructive success, providing a framework for personalized treatment that enhances surgical precision, predictability, and patient satisfaction [7,8,13,14]. Figure 2 provides a concise schematic of the Landfald–Olewnik classification used throughout this review. Each panel (a–e) represents a recurrent morphological archetype defined by belly configuration, insertion height, and the dominant contraction vector. The schematic is intended as a quick key for the clinical sections; detailed implications are discussed where relevant below, and we avoid repeating descriptive minutiae to keep the narrative focused.

### 1.4. Clinical Takeaway

The Landfald classification provides a systematic approach for describing ZMa/ZMi variants. Although derived from literature-based anatomical data, it offers valuable guidance for anticipating surgical challenges, tailoring procedural vectors, and improving aesthetic predictability. Integrating this classification into preoperative planning encourages accurate anatomical mapping and truly individualized intervention strategies. Despite detailed descriptions of facial mimetic anatomy, the procedural implications of zygomaticus variability remain underexplored.

## 2. The Significance of Zygomatic Muscles in Facial Aesthetics

### 2.1. Role in Contouring the Cheek and Malar Region

The ZMa and ZMi muscles are fundamental in shaping and maintaining the contours of the cheek and malar regions. Their anatomical position and dynamic activity influence both the static appearance of the midface and its dynamic transitions during expression [2,5]. Optimal muscle positioning and coordinated activation enhance malar projection, creating balanced midfacial contours that support facial harmony and perceived attractiveness [3,15].

Anatomical variability recently systematized in the Landfald classification, developed on the basis of a comprehensive literature review, demonstrates that deviations in belly configuration, insertion patterns, or symmetry can significantly impact the malar contour. Such variations may lead to aesthetic imbalance or asymmetry, even in the absence of overt pathology [11,12,13]. Awareness of these variants is essential for surgical and minimally invasive interventions, allowing clinicians to anticipate individual anatomical patterns and adapt procedural planning accordingly.

### 2.2. Clinical Takeaway—ZMa and ZMi in Midface Contouring

- Variants of ZMa and ZMi, as classified by Landfald, can significantly influence malar projection and facial symmetry.
- Recognizing these variants preoperatively allows adjustment of surgical and minimally invasive procedures for more predictable outcomes.

## 3. Procedural Implications of Zygomaticus Major and Minor Variability

### 3.1. Facelifting Techniques

Precise release of the ZMa is essential for natural midface repositioning. Multibellied or bifid variants may require targeted release, while low-insertion types often need a more vertical lift vector to prevent asymmetry and “joker smile” deformity [5,7,16,17]. Variants can also alter SMAS planes and ligament positions, requiring adjustment of surgical vectors to maintain symmetry [12,18,19,20].

### 3.2. Volume Restoration and Fillers

The ZMa delineates superficial and deep fat compartments, guiding filler and fat graft placement [7,14,19]. Bifid or accessory bellies may redirect filler diffusion and cause contour irregularities; dynamic US is recommended for detection [21,22,23]. Injection depth should be adapted: deeper bolus for accessory slips, subcutaneous placement for low-insertion ZMi.

### 3.3. Thread Lifting Techniques

Thread trajectories must align with native contraction vectors of ZMa and ZMi. Misalignment, particularly in bifid variants, risks asymmetry or visible thread tracks. US-guided planning improves safety and predictability [21,23,24].

### 3.4. Clinical Takeaway—Surgical and Minimally Invasive Planning

- Adapt facelift vectors when ZMa variants (bifid, multibellied, low-insertion) are present.
- Adjust filler planes to account for altered fat compartments or accessory slips.
- Use US guidance to plan thread trajectories and avoid asymmetry.

### 3.5. Clinical Risks and the Role of Anatomical Mapping

Anatomical variability of the ZMa and ZMi including differences in size, shape, insertion, and the presence of bifid or accessory bands is common and has direct consequences for aesthetic and reconstructive outcomes [14,25,26].

### 3.6. Key Variant-Related Risks

- “Joker smile”—lateral overcorrection from excessive traction, most often in low-insertion or bifid ZMa variants [5,7].
- Oral commissure retraction—due to asymmetrical muscular tension or misaligned thread placement, typically in asymmetrical type III or IV variants [16,17].
- Volume inconsistencies—uneven filler diffusion or contour defects caused by accessory bands, common in multibellied configurations [21,23].

### 3.7. Prevention and Mitigation

These complications are largely preventable through detailed preoperative mapping, combining:

- Dynamic US—to visualize belly configuration, insertion points, and compartment boundaries in real time.
- Electromyography (EMG)—to assess contraction vectors, asymmetries, and muscle hyperactivity.
- Integrated dynamic assessment—correlating imaging findings with functional expression to guide procedural planning [7,8].

Systematic use of US and EMG before facelifts, fillers, thread lifts, or reconstructive procedures allows the surgeon to adapt vectors, release patterns, injection planes, and thread trajectories to the patient’s unique mimetic anatomy, improving both safety and predictability.

### 3.8. Clinical Takeaway—Risk-Based Planning

- Match each ZMa/ZMi variant with its key procedural risk (e.g., “joker smile” in low-insertion types).
- Use US and EMG mapping before midface interventions to adapt surgical or injection techniques.
- Use dynamic assessment to bridge anatomical imaging with real-time functional behavior.

The clinical implications of specific ZMa/ZMi variants, their associated risks, and recommended technical adaptations are summarized in Table 1.

Anatomical variants of the ZMa and ZMi, including bifid, multibellied, low-insertion, and accessory-slip configurations can significantly affect both the safety and the predictability of aesthetic and reconstructive midface procedures. Linking each variant to its primary procedural risk and defining targeted technique adaptations enables surgeons to individualize their operative strategy. The following table integrates the Landfald classification with known clinical pitfalls and procedural modifications, providing a concise reference for surgical planning, filler injection, and thread-lift trajectory design.

## 4. Dynamic Assessment and Preprocedural Planning

### 4.1. Dynamic Assessment Tools: EMG, Ultrasound, and 3D Analysis

Preoperative mapping of the ZMa and ZMi using US, EMG, and three-dimensional (3D) facial expression analysis is essential for tailoring aesthetic and reconstructive interventions.

- US visualizes muscle boundaries, contraction vectors, and symmetry in real time, guiding filler placement, thread trajectory, and SMAS dissection while reducing procedural risk [22,23].
- EMG provides objective data on muscle activity during spontaneous or elicited expressions, detecting hyperactivity, weakness, or asymmetry [22,23].
- 3D analysis complements EMG by identifying functional imbalances missed on static assessment [3,21].

Integrating EMG with 3D analysis links objective contraction data to dynamic expression patterns, enabling surgical and injection vectors to align with each patient’s unique mimetic profile [3,21,23].

### 4.2. Patient Assessment Algorithm

A structured protocol improves safety and predictability in lifting, volumetric, and reconstructive procedures:

1. History—Prior surgery, trauma, or congenital anomalies affecting facial anatomy [2,7].
2. Visual inspection—Symmetry at rest and during expression; smile dynamics and midface mobility [1].
3. Functional palpation—Tone, contraction strength, symmetry, belly configuration, and accessory bands [12,20].
4. Imaging—US and EMG mapping; 3D analysis for complex or asymmetrical cases [3,21].
5. Classification—Classify ZMa/ZMi according to Figure 2 and record the panel letter (a–e) in the chart to guide vector planning and release path.
6. Integration—Combine anatomical and functional data to create a personalized procedural plan that preserves authentic facial expression [14,23].

### 4.3. Challenges in Integrating Functional and 3D Data

Synchronizing real-time muscle activation data (EMG, US) with static or semi-static 3D models remains technically challenging. This difficulty increases in the presence of variants such as bifid or accessory slips, which distort standard topography [12,20].

Validated, integrated multimodal platforms are needed to align physiological and anatomical data, enhancing surgical precision, reproducibility, and aesthetic fidelity [3,21].

### 4.4. Clinical Takeaway —Dynamic Assessment

- Integrate US, EMG, and 3D analysis to map contraction vectors and variant anatomy before planning interventions.
- A multimodal, standardized workflow improves safety, optimizes symmetry, and preserves natural expression.

The recommended preprocedural workflow for ZMa/ZMi-based midface procedures, integrating anatomical and functional assessment, is summarized in Table 2.

This workflow outlines the recommended preprocedural steps for aesthetic and reconstructive midface interventions involving the zygomaticus major (ZMa) and zygomaticus minor (ZMi). Each step integrates anatomical and functional assessment to ensure safety, optimize symmetry, and preserve authentic facial expression.

To translate these risks into procedure-specific actions, we provide a consolidated heat-map of risk categories and key adaptations (Table 3).

## 5. Smile Restoration: Adult and Pediatric Considerations

### 5.1. Muscle Transfer and Vector Reconstruction

Facial nerve paralysis disrupts midface symmetry by impairing the ZMa, the primary elevator of the oral commissure. Dynamic muscle transfer remains the gold standard for functional smile restoration [27,28].

- Gracilis transfer—preferred for its slender morphology, predictable contraction, and ease of microsurgical reinnervation; its oblique pull closely replicates the native ZMa vector [27].
- Masseter transfer—provides strong commissure elevation with native innervation but produces a more vertical vector requiring precise repositioning to mimic natural smile mechanics [27,28].
- Temporalis transfer—technically reliable and easily accessible but also generates a vertical lift and often requires extensive postoperative re-education [27,28]–Table 4.

Replicating the native ZMa contraction vector is critical for both functional and aesthetic success. Preoperative mapping with EMG, US, and 3D imaging supports accurate donor orientation, tension calibration, and minimization of postoperative asymmetry [3,22,23].

### 5.2. Pediatric Considerations in Congenital and Syndromic Cases

In pediatric craniofacial surgery, restoring natural midfacial dynamics requires detailed anatomical understanding of both ZMa and ZMi. This is essential in conditions such as cleft lip and palate, Treacher Collins syndrome, and hemifacial microsomia [12,28]

Congenital anomalies may produce aberrant muscle trajectories or absent insertions. If uncorrected, these defects can cause persistent asymmetry, impaired nonverbal communication, and psychosocial challenges [7,27].

Comprehensive preoperative evaluation integrating US, EMG, and dynamic facial analysis supports surgical planning that restores both functional biomechanics and emotional expressiveness [22,23].

### 5.3. Clinical Takeaway—Smile Restoration

- Gracilis transfer best replicates the native ZMa vector; masseter and temporalis require vector adjustment.
- Preoperative US/EMG/3D mapping ensures accurate donor orientation and tension calibration.
- In pediatric cases, early reconstruction should address both anatomy and emotional expressiveness to support psychosocial development.

## 6. Botulinum Toxin and Mimetic Precision

### 6.1. Botox in the Treatment of Gummy Smile

Accurate correction of a gummy smile requires understanding the anatomical variability of the ZMi and levator labii superioris muscles, which elevate the upper lip. Variations in attachment, insertion, and muscle volume can significantly alter treatment outcomes, necessitating personalized injection points and dosage strategies [16,17,21,29,30]. Preprocedural mapping (using US or EMG) improves precision, reduces risks of asymmetry, and ensures predictable aesthetic results [3,22,23].

### 6.2. Botox in the Treatment of Smile Asymmetry

In post-traumatic and post-surgical patients, altered muscle trajectories of the ZMa and ZMi require precise identification to restore balanced facial dynamics. Botulinum toxin can selectively relax hyperactive or misdirected fibers, but only if anatomical variability is carefully mapped. Misinterpretation of muscle courses risks worsening asymmetry or impairing facial function [12,31,32]. US and EMG are strongly recommended to guide individualized injection strategies and preserve natural expressions [3,23].

### 6.3. Clinical Takeaway—Botulinum Toxin for Midface Corrections

- Use US/EMG mapping to individualize injection sites and depth.
- Tailor dose and vector to minimize asymmetry or unnatural expression.
- Pay special attention to post-traumatic and post-surgical patients with altered muscle pathways.

## 7. Personalization of Aesthetic Procedures

### 7.1. Patient-Specific Mimetic Profile, Smile-Type Classification, and the Dynamic Aesthetics Paradigm

The concept of a *patient-specific mimetic profile* offers an advanced, individualized framework for facial aesthetic planning, emphasizing strategies tailored to the unique muscular anatomy and dynamic expression patterns of each patient. Given the considerable variability of the ZMa and ZMi muscles, such personalization is essential for achieving optimal aesthetic outcomes while preserving the natural dynamics of facial expression [3,32].

Establishing this profile begins with detailed anatomical and functional evaluation combining static and dynamic assessments to map muscle location, contraction vectors, and functional behavior. This enables precise customization of botulinum toxin injections, filler placement, and surgical techniques, minimizing the risk of asymmetry, unnatural expressions, and suboptimal results [12,14,21]

A key component of this approach is a preliminary classification of smile types based on biomechanics and morphology. Biomechanically, smiles may be predominantly horizontal, driven mainly by ZMa, or vertical/complex, influenced by ZMi and adjacent muscles such as the levator labii superioris and levator anguli oris [1,2,3]. Morphologically, classification can consider gingival display, oral commissure elevation, and symmetry, each shaped by the anatomical configuration and functional patterns of the zygomatic musculature [16,17].

This framework aligns with a broader paradigm shift in aesthetic medicine from a purely static approach—focused on soft tissue volume, wrinkle reduction, and contouring to dynamic aesthetics, which prioritizes preserving or enhancing natural facial movement. Muscles such as the ZMa and ZMi actively shape expressions, social interactions, and perceived attractiveness [1,25]. Neglecting these dynamic factors risks outcomes that appear anatomically correct but emotionally unconvincing. Modern planning therefore integrates dynamic evaluation tools—such as 3D imaging, US-guided mapping, and electromyography to ensure that structural enhancements harmonize with authentic facial expressions [3,12,23]. This dynamic-focused paradigm delivers results that are not only anatomically sound but also emotionally authentic and aesthetically compelling, enhancing both patient satisfaction and social perception outcomes.

### 7.2. Clinical Takeaway—Patient-Specific Mimetic Planning

- Classify smile type (horizontal vs. vertical/complex) and key features (gingival display, commissure elevation, symmetry) to guide planning.
- Use multimodal mapping (US, EMG, 3D) to tailor interventions to each patient’s mimetic profile.
- Apply the dynamic aesthetics paradigm to ensure outcomes that are both anatomically sound and emotionally authentic.

## 8. Dynamic Aesthetics and Smile Perception

### Zygomatic Muscles as Structures Influencing Movement Rather Than Just Volume

The ZMa and ZMi muscles are critical not only for providing midface volume and structural support but also for governing the dynamic aspects of facial movement and expression. Although volumetric augmentation strategies using fillers or fat grafting primarily address structural deficiencies, achieving aesthetically pleasing and natural results requires careful consideration of the functional dynamics and movement patterns influenced by these muscles [3,33]. The ZMa predominantly facilitates the characteristic oblique elevation of the oral commissure, essential for expressive smiling and emotional signaling, while the ZMi plays a subtler role in enhancing upper lip dynamics [6,25]. Thus, aesthetic procedures must not merely restore facial volume but also preserve or enhance natural muscular function, as impaired movement or unnatural muscular activation can lead to compromised aesthetic outcomes, including diminished authenticity and attractiveness of facial expressions [1,4,25]. To ensure optimal outcomes, clinicians are encouraged to integrate detailed anatomical and functional assessments, such as dynamic EMG and US imaging, into treatment planning, thereby promoting a balanced restoration of both facial volume and mimetic functionality [3,22,23].

## 9. Clinical Practical Recommendation

Anatomical and functional variability of the ZMa and ZMi muscles has direct implications for facial aesthetic and reconstructive procedures. Table 5 summarizes the most relevant risks and recommended adaptations.

### 9.1. Standardized Preoperative Assessment Protocol

1. Clinical history and inspection—document facial trauma, prior surgery, and baseline smile symmetry.
2. Functional palpation—assess tone, contraction strength, and presence of accessory bands.
3. Imaging—combine US for dynamic mapping, EMG for functional assessment, and MRI for deep-structure planning when complex anatomy is suspected.
4. Integration—correlate clinical and imaging findings to define anatomical variants.
5. Procedural planning—adapt lift vectors, filler planes, botulinum toxin sites, or graft orientation according to variant type.
6. Documentation and follow-up—record mapping findings and evaluate outcomes with repeat US/EMG at 1, 3, and 6 months.

### 9.2. Necessity for Preoperative Imaging

US provides real-time, cost-effective evaluation of ZMa/ZMi anatomy and contraction patterns, while MRI offers superior soft tissue contrast for complex or atypical cases. Integrating both modalities with EMG significantly enhances safety, precision, and personalization of midface procedures [3,22,23].

## 10. Conclusions

This review synthesizes current anatomical and clinical knowledge on the ZMa and ZMi muscles, highlighting their considerable morphological variability, symmetry patterns, and topographic relationships. Such variability present in both form and function has direct implications for aesthetic and reconstructive procedures, particularly those aiming to optimize midfacial dynamics and smile symmetry.

Accurate preoperative assessment should integrate traditional anatomical examination with advanced modalities such as US, EMG, and three-dimensional dynamic facial analysis. This combined approach enhances procedural precision, minimizes complications, and preserves the authenticity of facial expression.

Greater awareness of these anatomical nuances, along with further research into their surgical relevance, will support more predictable and individualized patient outcomes across diverse clinical contexts. Future validation of this workflow across clinical settings will further enhance safety, reproducibility, and training value in midface surgery.

## Figures and Tables

**Table 1:** Variant-specific risks and recommended technique adaptations.

Variant (Landfald Type)	Primary Risk	Recommended Technique Adaptation
Low-insertion ZMa	Lateral overcorrection (“Joker smile”)	Use more vertical lift vector; reduce lateral traction during facelifts
Bifid ZMa	Asymmetry, visible thread tracks	Adjust facelift/thread vectors to each belly; perform US mapping prior to dissection
Multibellied ZMa	Uneven filler diffusion, contour defects	Deeper bolus placement; modify injection planes based on compartment boundaries
Accessory slip ZMi	Perioral disharmony after augmentation	Shift injection depth to subcutaneous plane; adjust filler volume
Asymmetrical type III/IV ZMa	Oral commissure retraction	Reorient surgical or thread vectors; release tension asymmetrically

**Table 2:** Preprocedural workflow for midface procedures involving ZMa/ZMi.

Step	Action	Key Objectives	Tools/Methods
1	Clinical history	Identify factors affecting facial anatomy and neuromuscular function	Patient interview, medical records
2	Visual inspection	Assess symmetry at rest and in expression	Direct observation, photographic documentation
3	Functional palpation	Detect muscle tone, contraction strength, belly configuration, accessory bands	Manual palpation during facial movement
4	Imaging	Map anatomical course and functional dynamics	Ultrasound (US), Electromyography (EMG), 3D dynamic analysis
5	Classification (Figure 2 panel a–e)	Standardize variant labeling to guide vectors and release paths	Chart annotation linked to Figure 2
6	Integration	Correlate anatomical and functional data	Multimodal data review
7	Procedural execution	Apply variant-specific adjustments	Adapt vectors, injection planes, release strategy
8	Postoperative follow-up	Validate symmetry and function, detect complications early	Repeat US/EMG, photographic analysis

**Table 3:** Variant–risk–adaptation matrix for zygomaticus muscles. Risk coded as L(1)/M(2)/H(3). Procedures: facelift/midface, fillers (HA/Fat), thread lifting, BTX.

Variant\Procedure	Facelift/Midface	Fillers (HA/Fat)	Thread Lifting	BTX
**A. ZMa low-insertion (medial–inferior vector)**	**M(2)** • V-adj + RL-mod (limit vertical pull)	**H(3)** • Plane-Deep + Vol-control (modiolus area)	**H(3)** • NTZ at modiolus	**M(2)** • Dose-↓ + BTX-dist (spare ZMa)
**B. ZMa bifid**	**M(2)** • V-adj (more oblique vector)	**M(2)** • Plane-Deep	**M(2)** • NTZ over split zone	**M(2)** • BTX-dist (dominant branch)
**C. ZMa multibellied**	**M–H(2–3)** • RL-mod selectively	**M(2)** • Plane-Deep	**H(3)** • NTZ (avoid inter-belly septa)	**M(2)** • Dose-↓ (avoid mask-smile)
**D. Accessory slip to modiolus/upper lip**	**M(2)** • V-adj (more lateral vector)	**H(3)** • Plane-Deep + Vol-control	**H(3)** • NTZ near modiolus	**M(2)** • BTX-dist (target slip)
**E. ZMi hypertrophy/accessory band**	**L–M(1–2)** • V-adj (keep horizontal component)	**M(2)** • Plane-SMAS (shallower than ZMa-low)	**M(2)** • Careful over ZMi	**M(2)** • Dose-↓ on ZMi (avoid excessive down-pull)
**F. Asymmetry L/R**	**M(2)** • V-adj + RL-mod on dominant side	**M(2)** • Vol-control (asymmetric)	**M(2)** • Trajectory asymmetry	**M(2)** • Dose-asym (balance)
**G. High-insertion ZMa**	**L(1)** • Standard with slight correction	**M(2)** • Plane-SMAS	**M(2)** • Safer track (fewer conflicts)	**L–M(1–2)** • Standard/↓ dose
**H. Zygomaticus–LLS complex variant**	**M–H(2–3)** • RL-mod (control vertical component)	**M(2)** • Plane-Deep	**M(2)** • Avoid crossing LLS line	**M(2)** • BTX-dist (balance ZMa/ZMi/LLS)
Legend:	L(1) Low risk	M(2) Moderate risk		H(3) High risk

**Abbreviations:** V-adj, vector adjustment; Plane-Deep/SMAS/S, target plane; NTZ—no-thread zone; RL-mod, retaining ligament release modification; BTX-dist, tailored botulinum toxin distribution; Dose—↓/↑, dose trend; Vol-control, volume control; LLS—levator labii superioris.

**Table 4:** Comparison of muscle transfer techniques for smile restoration.

Donor Muscle	Vector Orientation vs. Native ZMa	Advantages	Limitations	Optimal Use Cases
Gracilis	Oblique pull closely replicates ZMa vector	Slender morphology; predictable contraction; ease of microsurgical reinnervation	Requires microsurgical expertise; donor site morbidity	Gold standard for dynamic smile restoration; best vector match
Masseter	Predominantly vertical; requires repositioning	Strong elevation; native innervation; reliable performance	Less natural smile mechanics without repositioning	Patients prioritizing strength over vector precision
Temporalis	Predominantly vertical; requires repositioning	Technically reliable; accessible donor site	Requires extensive re-education; less natural vector	When gracilis or masseter unavailable or contraindicated

**Table 5:** Clinical implications of ZMa/ZMi variability in aesthetic and reconstructive procedures.

Procedure	Risk Due to Anatomical Variation	Adaptation of Clinical Technique
Facelift / Midface Lift	Asymmetry; inappropriate vector tension; ‘joker smile’ deformity	Preoperative mapping of ZMa/ZMi course; modify lifting vectors
Soft Tissue Augmentation (HA, Fat Grafting)	Uneven filler distribution; asymmetry due to additional bands	US-based mapping; adjust injection planes and volumes
Thread Lifting	Thread misplacement; insufficient lift from unexpected muscle paths	Preoperative imaging and palpation; individualized thread planning
Botulinum Toxin Therapy	Incorrect toxin placement; asymmetric smile or incomplete correction	Variant-specific mapping; adjust injection points and dosing
Smile Reconstruction Surgery	Failure to restore natural smile dynamics	Customized graft placement respecting contraction vectors
Pediatric Craniofacial Surgery	Long-term deficits due to abnormal muscle topography	Surgical recreation of normal ZMa/ZMi arrangement

## Data Availability

Not applicable.
